# Location Determination of Impact on the Wind Turbine Blade Surface Based on the FBG and the Time Difference

**DOI:** 10.3390/s21010232

**Published:** 2021-01-01

**Authors:** Bingkai Wang, Wenlei Sun, Hongwei Wang, Yunfa Wan, Tiantian Xu

**Affiliations:** School of Mechanical Engineering, Xinjiang University, Urumqi 830047, China; wangbk@stu.xju.edu.cn (B.W.); wanghongwei@stu.xju.edu.cn (H.W.); wanyunfa@stu.xju.edu.cn (Y.W.); xutiantian@stu.xju.edu.cn (T.X.)

**Keywords:** fiber Bragg grating (FBG), damage detection, wind turbine blade (WTB), impact location, time difference, variational mode decomposition (VMD)

## Abstract

This paper proposes an approach to the determination of the precise location of an impact on the surface of a wind turbine blade (WTB) based on a fiber Bragg grating (FBG) and the time difference, and its effectiveness is verified by experiments. First, the strain on the WTB surface is detected with an FBG. Then, the signal is decomposed into a series of components via variational mode decomposition (VMD), and some signals with impact characteristics are chosen for reconstruction. The instant energy of the reconstructed signal is then amplified through the Teager energy operator (TEO) to identify the time difference between FBGs. Finally, the coordinate of the impact point is obtained by solving the hyperbolic mode with the time difference. The results of experiments demonstrate that the proposed approach exhibits good performance with high accuracy (97%) and low error (12.3 mm).

## 1. Introduction

Blades are the most important component of wind turbines for wind energy conversion. However, factors such as their harsh installation environment and the increasing size of wind turbines have increased the probability of impacts from birds, hail, and flying rocks. The defects and damage of the wind turbine blade (WTB) not only reduce its service life and power generation rate, but also increase safety risks and maintenance costs [1]. Therefore, the research on the technology for the location determination of impacts on the WTB surface is of great significance for damage detection and risk reduction. Some current detection technologies, including noise analysis [2,3], vibration analysis [4,5], acoustic emission technology [6,7], and image recognition [8,9], have been vigorously investigated for the damage detection of WTBs.

In addition, due to their light structure, high signal-to-noise ratio, and strong reusability, fiber Bragg grating (FBG) has successfully been applied to the strain monitoring of various structures [10]. Matveenko et al. [11] proposed a method of quoting strain values between FBG sensors at different positions and carried out tensile experiments on glass fiber samples, realizing a rough judgment of the damage location. Jin et al. [12] studied the relationship between the FBG wavelength migration and the crack growth length and monitored the crack growth on an aluminum alloy plate using the FBG reflection spectrum. Yu et al. [13] constructed an FBG sensor network to address the problem of low-speed impact location determination. A reliable method for the determination of an impact location on carbon fiber composite panels was provided based on the skewness and steepness of the signal time series. Alvarez et al. [14] developed a damage diagnosis method based on strain field pattern recognition with FBG detection technology and obtained good results in experiments conducted on wings. Sierra et al. [15] developed a feature recognition method based on the strain fields measured by different sensors using optical fiber sensing technology. Defects were detected in an experiment on a damaged WTB structure, and the sensitivity of the technology was evaluated.

A new method of adaptive signal processing, namely variational mode decomposition (VMD), is widely used in the field of fault detection due to its good performance and robustness. Ma et al. [16] proposed a method that combines VMD and the Teager energy operator (TEO) to overcome the low signal-to-noise ratio and difficulty in extracting fault features of rolling bearings and verified its effectiveness via experiments. Li et al. [17] proposed an early detection method for flutter based on VMD and the differential power spectrum entropy, and experiments revealed that this method has important practical application value. Li et al. [18] used VMD to divide a signal into overlapping frames during the milling process, and selected scale-sensitive features for online vibration detection. The analysis results revealed that the proposed method could effectively detect chatter vibration under both stable and variable cutting conditions. However, VMD has rarely been employed in existing research on WTB impact detection.

The main problem in the current research on the location determination of impacts on WTBs is that the location accuracy is not high enough, and most of the results represent cursory locations. To solve this problem, in the present study, VMD is innovatively introduced into the field of WTB monitoring. A coordinate calculation approach to precisely locate impacts on a WTB is proposed to improve the location accuracy. First, an efficient signal feature extraction method is developed to ensure the location accuracy from the perspective of parameter sources. Second, a mathematical model of location calculation is established and optimized. Finally, an experiment on locating impact on a WTB surface is carried out, and the effectiveness of the proposed approach is evaluated via the experimental results.

## 2. Proposed Approach

Determining the location of an impact on the WTB surface can be considered as a problem that can be solved by a mathematical model, and consists of three phases, namely, strain signal acquisition and preprocessing, feature parameter extraction, and the calculation of the solution.

### 2.1. Strain Signal Acquisition and Preprocessing

The WTB is an elastic solid medium. When a certain point on the surface of the WTB receives an impact, the WTB can be regarded as undergoing transient excitation. The vibration energy of any mass point will be transferred to nearby mass points due to mutual cohesion, which will cause the vibration of the nearby mass points. The particle vibration propagates the changes of stress and strain to the surrounding areas in the form of waves in the elastic medium. This wave is called a stress wave [19].

**Figure 1:**
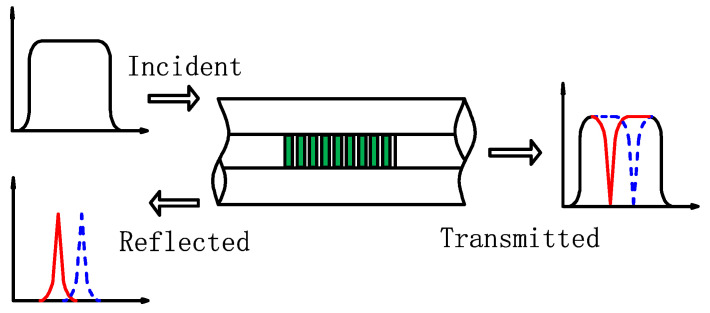
Principle of fiber Bragg grating (FBG).

In this study, FBG sensors were used to detect the strain state on the WTB surface to obtain strain signals, and the detection principle is presented in Figure 1. The FBG is a grating structure with the periodic perturbation of refractive index in the fiber core. The central wavelength of the reflected light, which is generated due to the grating, is affected by the grating period and the effective refractive index of the fiber core. The change of the externally measured quantities (e.g., strain, temperature, etc.) will cause the axial deformation of the FBG, thereby causing the wavelength migration of its reflected light. The relationship between axial strain and wavelength migration is expressed as

(1) $$\Delta \varepsilon =\frac{1}{\lambda_{B}\left(1-P_{e}\right)}\Delta \lambda$$

where Δε is axial strain, Δλ is wavelength migration, $\lambda_{B}$ is the original central wavelength of the FBG, and $P_{e}$ is the elastic-optical coefficient of the fiber material. The theory reflects the transfer relationship between the migration of the reflected wavelength and the measured strain change of the FBG, which can provide theoretical guidance for the practical application of the FBG. Therefore, the wavelength migration of the FBG can be considered as the characteristic carrier signal of the WTB to reflect the strain change in the test area. 

Generally, the impact response signal contains a relatively large direct-current component (DC) interference component, which will affect the signal-to-noise ratio of the system and subsequent signal analysis; thus, the original signal must be processed to remove the DC component [20]. The arrival time of the impact signal is roughly estimated after removing the DC component, and a fuzzy interval including the arrival time of the impact signal is intercepted for subsequent processing to reduce the calculation amount.

### 2.2. Feature Parameter Extraction

In addition to the impact signal, the strain signal of the WTB measured by FBG sensors includes other complex components, such as structural noise and system noise. The impact signal refers to the strain-change signal directly caused by the impact, which is useful for determining the locations of impacts. Because the impact is a short transient process, the impact signal must have the characteristic of a short action time, and its frequency is usually concentrated in a higher range in the frequency spectrum. In addition, relative to the larger structure of the WTB, the strain value caused by impact should not be huge; although it is obvious, it should be less than the strain value caused by the structural resonance of the WTB itself.

Structural noise mainly refers to the strain signal formed by the repeated storage and superposition of strain energy after the WTB is impacted due to the structural resonance of the WTB itself [21]. Although the formation process of structural noise signals is complicated, they still present obvious characteristics, such as a long action time, large strain amplitude, and low range of frequency distribution. The presence of structural noise will interfere with the extraction of the feature parameters of the effective signal and will then affect the accuracy of the final impact location determination.

System noise refers to the noise interference signal that exists in the entire detection system, including the WTBs, detection devices, and power supply devices. The sources of this type of signal are heterogeneous and difficult to completely remove, even if the signal has previously been processed to remove the DC component. The characteristics of the system noise signal are that it always exists before and after impact, its amplitude is small, and its frequency is very high. The characteristics of the various types of signals will facilitate the identification of impact signals and the extraction of feature parameters. The feature parameter to be extracted in this approach is the moment at which the impact signal arrives at the FBG sensor, namely, the wave arrival moment (WAM).

#### 2.2.1. Identification of the Impact Signal with VMD

To obtain the impact signal from the complex broadband signal superimposed by multiple signals, the signal is first decomposed and processed by VMD. The VMD algorithm decomposes the original input signal f into *K* intrinsic mode function (IMF) components with different center frequencies and bandwidths by constructing and solving constrained variational problems, thereby effectively separating each frequency component of the signal. The process is outlined as follows.

(1) Hilbert transform is performed on each IMF component to obtain its analytical signal.
(2) The center frequency of each analytical signal is estimated, and the frequency spectrum of each analytical signal is modulated to the corresponding baseband via frequency-shifting.
(3) The Gaussian smoothing index of the frequency-shifted signal is used to estimate the bandwidth of each IMF component to obtain a constrained variational model. The model is expressed as
  (2) $$\{\begin{array}{l} \underset{\left\{u_{k}\right\},\left\{\omega_{k}\right\}}{\min}\left\{\sum_{k}{\left\|\partial_{t}\left[\left(\delta \left(t\right)+\frac{j}{\pi t}\right)u_{k}\left(t\right)\right]e^{-j\omega_{k}t}\right\|}_{2}^{2}\right\}\\ s.t\sum_{k}u_{k}=f \end{array},$$
  where $\left\{u_{k}\right\}=\left\{u_{1},\cdots ,u_{K}\right\}$ indicates the *K* IMF components obtained by decomposition, and $\left\{\omega_{k}\right\}=\left\{\omega_{1},\cdots ,\omega_{K}\right\}$ is the center frequency of each component.
(4) The quadratic penalty function term and the Lagrange multiplier are used to transform Equation (2) into an unconstrained problem expressed as
  (3) $$\begin{array}{l} L\left(\left\{u_{k}\right\},\left\{\omega_{k}\right\},\lambda \right)=\\ \alpha \sum_{k}{\left\|\partial_{t}\left[\left(\delta \left(t\right)+\frac{j}{\pi t}\right)u_{k}\left(t\right)\right]e^{-j\omega_{k}t}\right\|}_{2}^{2}+{\left\|f\left(t\right)-\sum_{k}u_{k}\left(t\right)\right\|}_{2}^{2}+\langle \lambda \left(t\right),f\left(t\right)-\sum_{k}u_{k}\left(t\right)\rangle \end{array}$$
  where α is a penalty factor and λ(t) is the Lagrange multiplier. The final solution is obtained by the alternate direction method of multipliers, and the adaptive decomposition of the frequency band is realized [22].

From the obtained multiple groups of IMF signal components with different center frequencies, one or more groups with similar characteristics as the impact signal are selected for reconstruction to obtain signals with relatively simple structural components. This signal, excluding other interference and noise signals, has the same characteristics as the signal caused by the impact, and can be regarded as a pure impact signal.

#### 2.2.2. Demodulation of the Signal with the TEO

To obtain more obvious time-series features and accurately extract the WAM, the TEO is selected to demodulate the signal. The TEO, a nonlinear difference operator, can enhance the instantaneous energy characteristics of time-series signals via the nonlinear combination of the instantaneous value of the signal and its differential, and is suitable for the detection of the impact component in signals. The principle is summarized as follows.

- Define Ψ as a TEO; for any continuous impact response signal, it is defined as
  (4) $$\Psi \left[x\left(t\right)\right]={\left[\dot{x}\left(t\right)\right]}^{2}-x\left(t\right)\ddot{x}\left(t\right)$$
  where $\dot{x}\left(t\right)$ and $\ddot{x}\left(t\right)$ are respectively the first- and second-order integrals of the impact response signal x(t). However, the signals collected in practice are mostly discrete. Equation (4) is then discretely converted to obtain Equation (5), which is given as follows.
  (5) $$\Psi \left[x\left(n\right)\right]={\left[x\left(n\right)\right]}^{2}-x\left(n-1\right)x\left(n+1\right)$$
- The characteristic signal after VMD and reconstruction, described in Section 2.2.1, is introduced into Equation (5) for calculation, and an impact signal with an enhanced instantaneous energy feature is obtained. The signal has simple components and an obvious instantaneous energy feature in the time series and can therefore be directly used for the extraction of the WAM. The method of setting a threshold is adopted during the WAM extraction process. As the strain values detected by FBG sensors in different positions will be different, the energy of each group of signals will be different, and it is therefore difficult to set the threshold uniformly. Thus, the extreme value normalization formula given by Equation (6) is used to dimensionalize the data so that each group of signals is unified between 0 and 1.
  (6) $$x^{\prime }=\frac{x-x_{\min}}{x_{\max}-x_{\min}}$$
  where x is the primary data, $x_{\max}$ is the maximum primary data, and $x_{\min}$ is the minimum primary data. When the transient energy amplitude of the impact signal exceeds the set threshold for the first time, the corresponding time point is extracted, which is the WAM.

### 2.3. Calculation of the Solution

A method of impact location determination using the time difference is proposed based on the relationship between the propagation distance of the impact signal and its propagation velocity and time. Two assumptions must be made when calculating the position of an impact on the WTB surface via the time-difference method: (1) the surface of the WTB is approximately regarded as a two-dimensional plane space, and all calculation points fall within the plane space; and (2) the material is isotropic, and the propagation velocity of the stress wave is constant throughout the WTB surface. 

**Figure 2:**
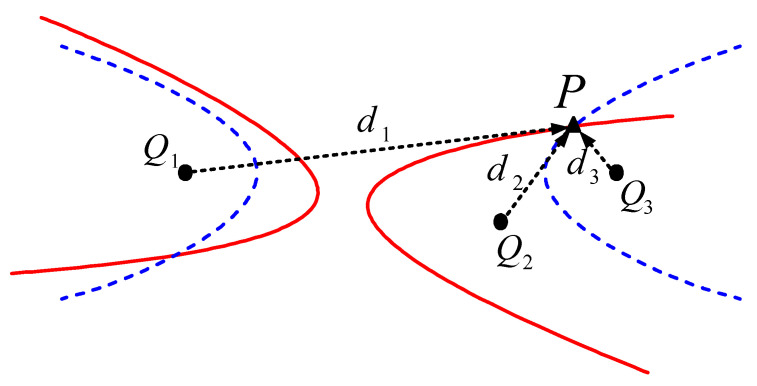
Principle of impact location determination based on hyperbolic equations.

Based on the assumptions, a plane rectangular coordinate system, for which a point close to the root of the WTB is selected as the coordinate origin, can be established on the WTB surface. The position of each FBG applied on the WTB surface is defined as $Q_{1}$, $Q_{2}$, $Q_{3}$…, and the coordinates are respectively $Q_{1}\left(x_{1},y_{1}\right)$, $Q_{2}\left(x_{2},y_{2}\right)$, $Q_{3}\left(x_{3},y_{3}\right)$… The impact position is point P(x,y), and the propagation time of the stress wave from *P* to each *Q* is respectively $t_{1}$, $t_{2}$, $t_{3}$… which is numerically equal to the WAMs extracted in Section 2.2.2. One hyperbolic equation with two *Qs* as the focus can be established from one time difference between any two WAMs. Therefore, at least two time differences, namely three basis points, are required to establish two hyperbolic equations. The intersection of two hyperbolic equations is the position at which the WTB surface is impacted. Figure 2 presents the principle of location determination based on the hyperbolic equations, and the mathematical model is expressed as Equation (7):

(7) $$c\;\Delta_{ij}=d_{i}-d_{j}=\sqrt{{\left(x-x_{i}\right)}^{2}+{\left(y-y_{i}\right)}^{2}}-\sqrt{{\left(x-x_{j}\right)}^{2}+{\left(y-y_{j}\right)}^{2}}$$

where c is the propagation velocity of the stress wave on the WTB surface, $\Delta_{ij}$ is the propagation time difference between any two WAMs, $\Delta_{ij}=t_{i}-t_{j}$, and d is the distance from the impact point to each *Q*; i,j=1,2,3⋯

It is worth noting that there are usually two intersection points obtained by solving two hyperbolic equations. To solve this problem, the solution process of the hyperbolic equations is improved to make the solution unique. In fact, from the perspective of combinatorial mathematics, three hyperbolic equations with unique solutions can be established via three *Q* and are respectively expressed as follows.

(8) $$c\;\Delta_{12}=\sqrt{{\left(x-x_{1}\right)}^{2}+{\left(y-y_{1}\right)}^{2}}-\sqrt{{\left(x-x_{2}\right)}^{2}+{\left(y-y_{2}\right)}^{2}}$$

(9) $$c\;\Delta_{13}=\sqrt{{\left(x-x_{1}\right)}^{2}+{\left(y-y_{1}\right)}^{2}}-\sqrt{{\left(x-x_{3}\right)}^{2}+{\left(y-y_{3}\right)}^{2}}$$

(10) $$c\;\Delta_{23}=\sqrt{{\left(x-x_{2}\right)}^{2}+{\left(y-y_{2}\right)}^{2}}-\sqrt{{\left(x-x_{3}\right)}^{2}+{\left(y-y_{3}\right)}^{2}}$$

The division and transformation of Equations (8–10) yield the following.

(11) $$\{\begin{array}{c} \frac{\Delta_{12}}{\Delta_{13}}=\frac{\sqrt{{\left(x-x_{1}\right)}^{2}+{\left(y-y_{1}\right)}^{2}}-\sqrt{{\left(x-x_{2}\right)}^{2}+{\left(y-y_{2}\right)}^{2}}}{\sqrt{{\left(x-x_{1}\right)}^{2}+{\left(y-y_{1}\right)}^{2}}-\sqrt{{\left(x-x_{3}\right)}^{2}+{\left(y-y_{3}\right)}^{2}}}\\ \frac{\Delta_{12}}{\Delta_{23}}=\frac{\sqrt{{\left(x-x_{1}\right)}^{2}+{\left(y-y_{1}\right)}^{2}}-\sqrt{{\left(x-x_{2}\right)}^{2}+{\left(y-y_{2}\right)}^{2}}}{\sqrt{{\left(x-x_{2}\right)}^{2}+{\left(y-y_{2}\right)}^{2}}-\sqrt{{\left(x-x_{3}\right)}^{2}+{\left(y-y_{3}\right)}^{2}}} \end{array}$$

**Figure 3:**
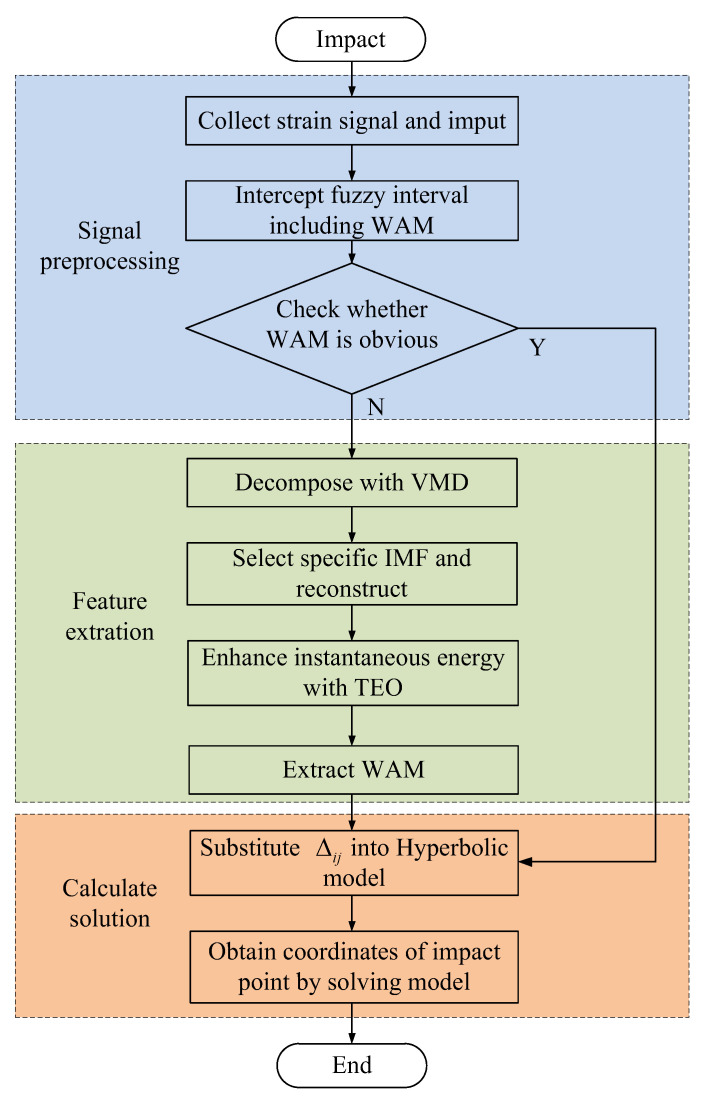
Flow diagram of the proposed approach.

The solution of Equation (11) can be obtained as

(12) $$\left(x,y\right)=F\left(\Delta_{12},\Delta_{13},\Delta_{23},x_{1},y_{1},x_{2},y_{2},x_{3},y_{3}\right)$$

where F is the operation function of the solution. It is evident that the solution of the model is independent of the propagation velocity of the stress wave, and only the WAM is required to obtain the coordinates of the position of the impact on the WTB surface. Therefore, the impact position coordinates can be obtained by substituting the extracted WAMs into Equation (12) and inputting the *Q* coordinate parameters of the corresponding FBG. At this point, the location of the impact on the WTB surface is completed. The flow diagram of the proposed approach is presented in Figure 3.

## 3. Experiment and Discussion

### 3.1. Impact Location Experiment

To verify the effectiveness of the proposed approach, an impact location determination experiment on a WTB surface was carried out. The experimental object was a real WTB model scaled down from an actual WTB, the material of which was glass fiber. The material performance and size parameters are reported in Table 1, and the natural frequency of the WTB is exhibited in Table 2.

**Figure 4:**
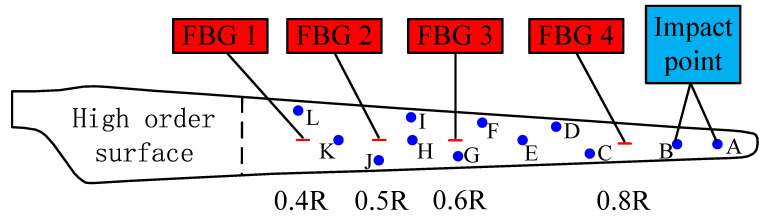
Location distribution of the FBGs and impact points.

**Figure 5:**
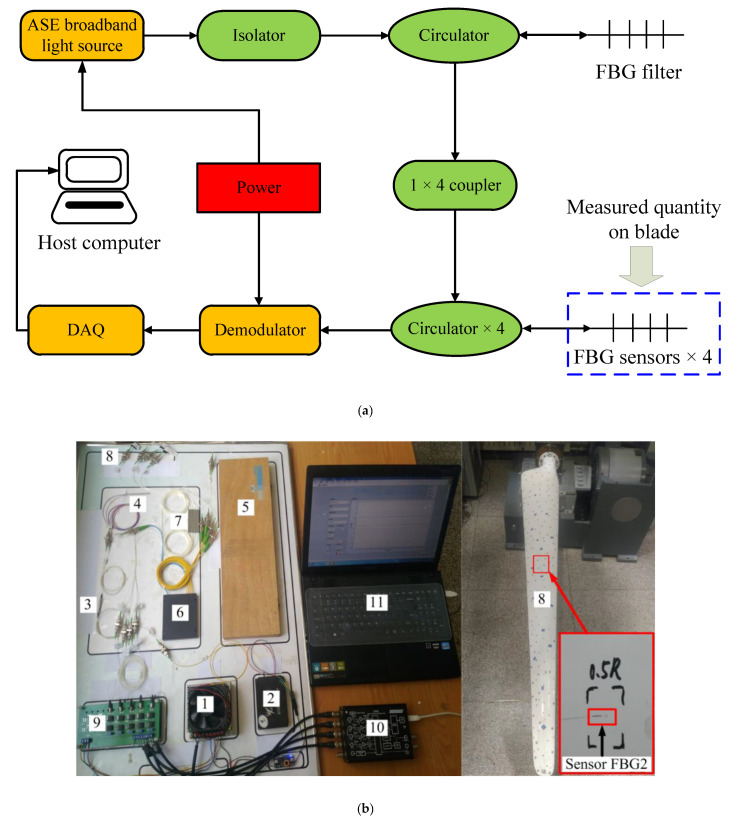
(**a**) FBG detection systems; (**b**) Experimental set-up. 1—power; 2—Amplified Spontaneous Emission broadband light source; 3—isolator; 4—circulator; 5—FBG filter; 6—coupler; 7—circulator; 8—FBG sensors; 9—demodulator; 10—Data Acquisition; 11—host computer.

The WTB was fixed on a support bench in the form of an end constraint, and the full length of the WTB from the root to tip is defined as *R*. The surface of the WTB near the root area had the geometric characteristics of a high-order curved surface with greatly variable curvature, and it was difficult to be regarded as a two-dimensional plane space. The WTB from 0.3*R* to *R* was selected as the effective experimental length range. Figure 4 presents the application positions of the FBGs and the impact point distribution in the experiment. Four FBGs, namely FBG1, FBG2, FBG3, and FBG4, were respectively glued at 0.4*R*, 0.5*R*, 0.6*R*, and 0.8*R* along the WTB span direction by epoxy resin adhesive (α-cyanoacrylate), which was selected due to its strong adhesion (18–26 MPa), short curing time (60–90 s) and wide application temperature (−50 to 120 °C). In this experiment, the FBGs were customized, and its main characteristics are reported in Table 3. The sensitivity of FBG interrogator is 1.22 pm/με and the resolution is 0.23 pm. Twelve impact test points (respectively denoted as impact points *A*–*L*) scattered on the WTB surface included all the relative positional relationships with the FBGs, ensuring the universality and reliability of the experimental results. Figure 5 presents the composition of the FBG detection system and the site of the impact location determination experiment. First, an impact load was applied to one of the impact points. Then the strain data collected by FBGs were recorded. Finally, the data were processed and calculated according to the approach proposed above. The accuracy of the proposed approach was verified by comparing the detection result with the actual location of impact point. To ensure the reliability of the experiment, at least 10 sets of tests were carried out on each of the 12 impact points during the experiment. Each set of data was collected for about 17 s, and the sampling frequency was 10,000 Hz. In this experiment, the impact was generated by a ball hitting the surface of blade. The energy of impact was controlled by the mass of the ball and the height of ball’s free fall.

### 3.2. Results and Discussion

**Figure 6:**
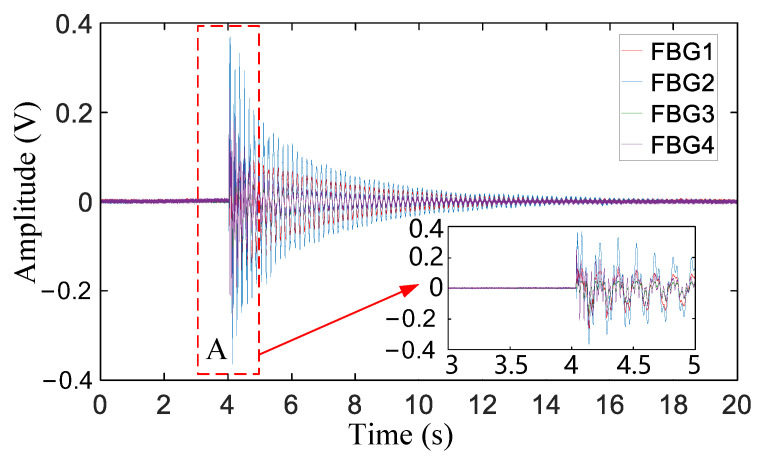
Impact signal after removing the DC component.

Taking impact point A as an example, Figure 6 presents the signals collected by each FBG after removing the DC component. It is evident that the impact signal arrived at approximately 4 s, and the four groups of signals all exhibited a damping attenuation trend after a sudden change in amplitude. This process indicates that the WTB was subjected to damping vibration due to its own damping effect after the impact. In addition, the structural characteristics of the WTB explain the phenomenon of the magnitude of FBG2 being larger than the magnitudes of the other FBGs. Only the ambiguous interval of the WAM could be obtained from the signal shown in the enlarged view at impact point A, meaning that the WAM of each group of signals could not be directly and accurately determined. To reduce the amount of subsequent calculation, a total of 2 s (from 3 to 5 s) were intercepted as the fuzzy interval of the WAM, and subsequent processing was performed on the signal in the fuzzy interval.

**Figure 7:**
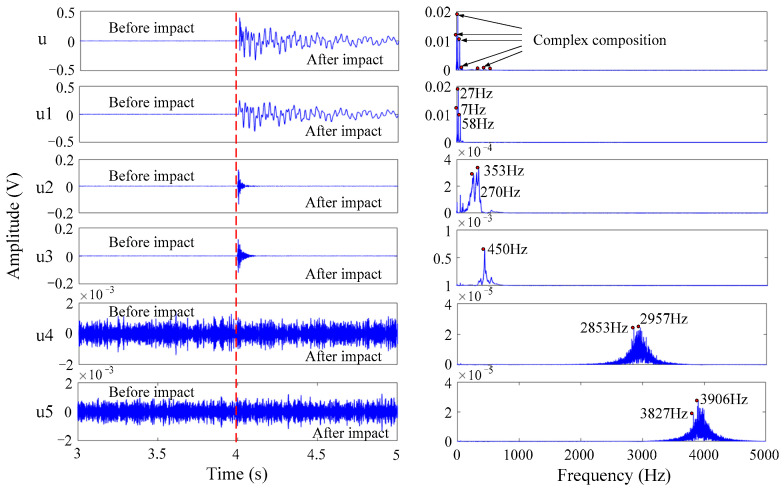
Results of the variational mode decomposition (VMD) of FBG2.

Figure 7 exhibits the results of the VMD of the impact signal of FBG2 in the fuzzy interval. In the figure, component *u* (left) is the initial signal before the VMD of FBG2, and component *u* (right) is the corresponding spectrum. It is evident that component *u* contained complex signal components. Components *u*1–*u*5 are respectively five groups of IMFs with different center frequencies and their corresponding frequency spectra after VMD decomposition. After analysis, it was found that component *u*1 reflected the features of a longer action time, larger amplitude, and a center frequency mainly concentrated below 100 Hz. As a result, with reference to Table 2, component *u*1 was an interference signal, the main component of which was the structural resonance of the WTB. The frequencies of components *u*4 and *u*5 were too high, the amplitudes were too low, and the action times covered the entire process before and after the impact. They were typical interference signals with system noise as the main component. Components *u*2 and *u*3 had shorter action times and higher frequencies, which conforms to the short-term and high-frequency vibration characteristics of transient impact. Consequently, components *u*2 and *u*3 were selected as the target signal components for subsequent reconstruction, processing, and analysis.

**Figure 8:**
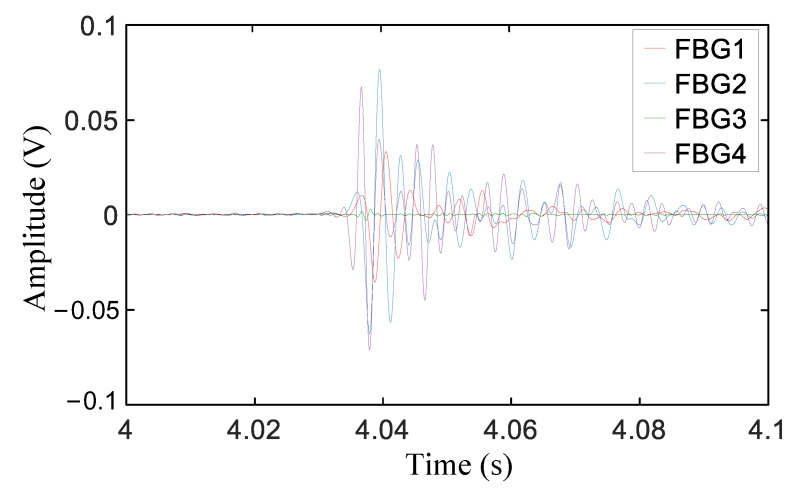
The reconstructed signals.

The reconstructed signals of FBG1, FBG2, FBG3, and FBG4 are shown in Figure 8. Via comparison with Figure 6, it can be seen that the time-series features of the signals after VMD and reconstruction were clearer, and the degree of time recognition was significantly improved. However, the moment corresponding to the highest peak of each group of signals lagged behind the real WAM. Moreover, the hysteresis ratio changed due to the different propagation distances of the stress wave, which affected the accuracy of WAM extraction; therefore, it is not suitable to select the highest peak value as the feature for WAM extraction. In addition, the amplitude of the first wave was too weak to judge the starting state, and the signal was easily submerged by noise. Therefore, it is unrealistic to find the WAM by directly using the first wave. 

**Figure 9:**
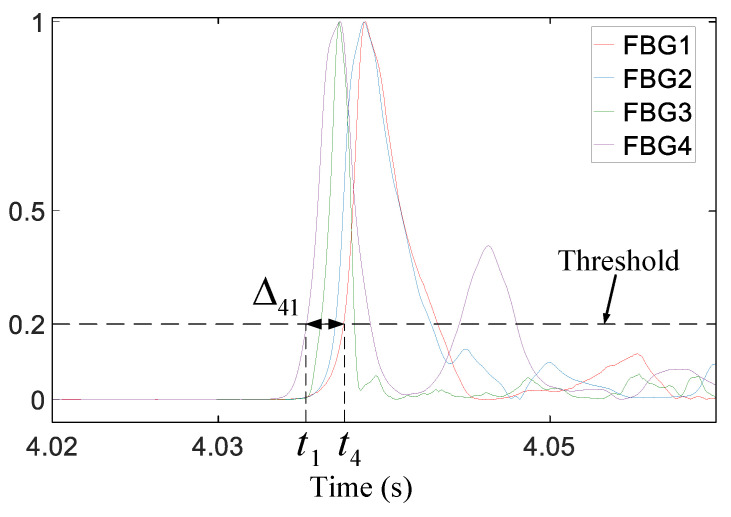
Time difference of the instant Teager energy.

**Figure 10:**
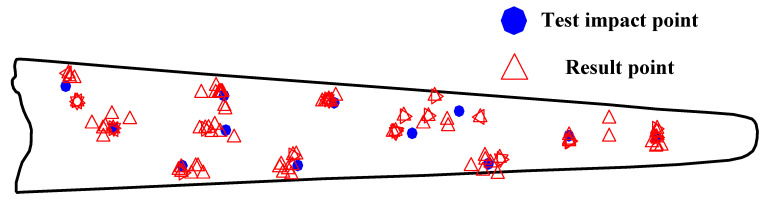
Results of the impact location determination experiment.

As shown in Figure 9, the reconstructed signal was demodulated with the TEO and then normalized. Because the instantaneous energy of the time-series feature was significantly enhanced, the first wave of the impact signal could be easily found. When the instantaneous energy of the signal exceeds the set threshold for the first time, the WAM can be accurately extracted and further used to calculate the time difference. All groups of time differences extracted according to the method described in Section 2.3 were introduced into Equation (12) to obtain the impact position, and the calculation results are presented in Figure 10. It can be qualitatively and intuitively evaluated that almost all the impact positions obtained via the proposed approach fell near the actual corresponding impact point, and the effect of the experiment was good.

**Figure 11:**
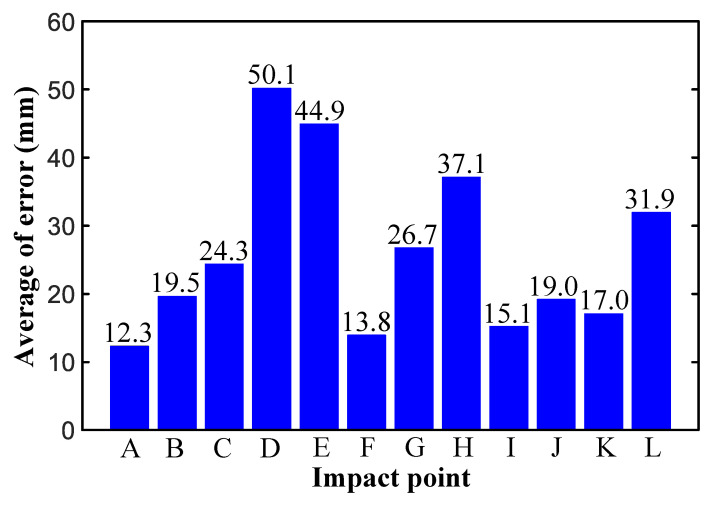
The mean absolute error at the impact points.

**Figure 12:**
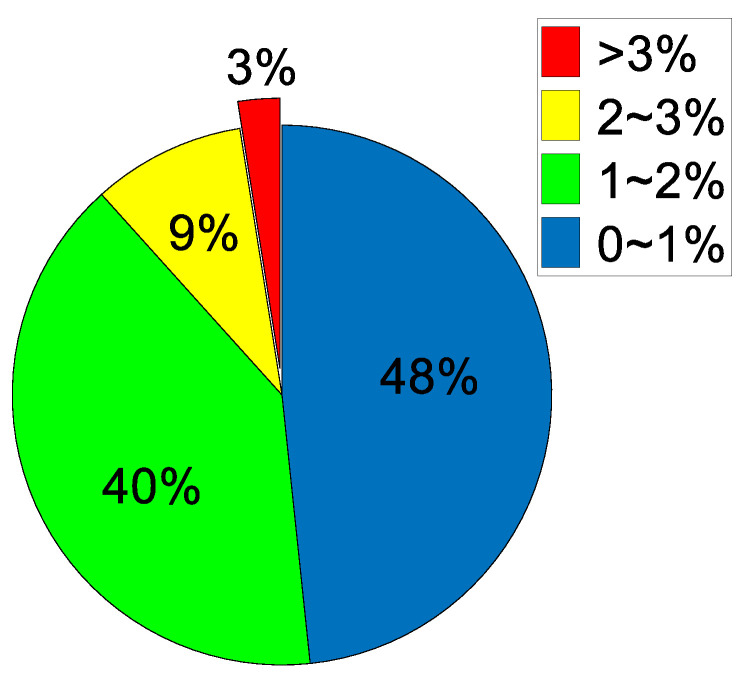
Proportions of relative error.

To conduct a better quantitative evaluation of the test results, two evaluation concepts are introduced: (1) the absolute error is defined as the distance between the positions of the calculated and real impact points, and (2) the relative error is defined as the ratio of the absolute error to the total length of the WTB in the span direction. The mean absolute error for each of the 12 impact points was calculated after eliminating the extreme value. The mean absolute error of most of the detection results was less than 30 mm, that of point D was the maximum (50.1 mm), and that of point A was the minimum (12.3 mm). The error distribution exhibited no obvious law, as shown in Figure 11. A statistical chart of the relative error proportions of all data is shown in Figure 12, from which it is evident that, of all the experimental data, only 3% of the test results had a relative error greater than 0.03, and those with a relative error of less than 0.02 accounted for nearly 90%. 

For all 12 impact test points, the average absolute error of the locating results was 26.0 mm, and the relative error was 1.1%. While the relative error that was obtained by method based on the skewness and steepness was bigger than 8% in Yu’s research [13], Staszewski [23] obtained a relative error of 1.3% via modified triangulation procedure with genetic algorithms. The comparison of results therefore reflect higher accuracy of proposed approach in this research.

After analysis, the error was found to primarily originate from the following two aspects: (1) the real surface of the WTB was an approximate plane surface, which affected the location calculation accuracy to a certain extent, and (2) the recognition of time had a limitation, which caused a certain error in the extraction of the WAM due to the limited sampling frequency in the experiment.

## 4. Conclusions

In this study, an approach to the precise determination of the location of an impact on the surface of a WTB based on FBG and the time difference was proposed. In the proposed method, a rectangular coordinate system is established on the WTB surface based on the plane assumption, and the mathematical hyperbolic model for locating the impact is established, the solution process of which is improved. In this way, complex physical problems are transformed into simple mathematical parameter-solving problems, which provides new ideas for the precise determination of the locations of impacts on the WTB surface.

Combined with the TEO, the VMD algorithm was innovatively introduced to the strain detection of the WTB surface to enhance the instantaneous energy feature of the signal and improve the time recognition of the impact signal. Via this method, the high accuracy of impact location calculation in the data source is ensured.

According to the proposed method, an experiment of the location determination of impacts on a WTB surface was carried out. The results revealed that the relative error was less than 0.03, and a high accuracy of 97% was therefore achieved. One advantage of this approach is that feature training with a large amount of prior data is not required. Moreover, this method has universal applicability in different environments with different states and models. 

## Figures and Tables

**Table 1:** The parameters of the wind turbine blade (WTB).

Density (kg·m^−3^)	Length (m)	Poisson’s Ratio	Shear Modulus (MPa)	Young’s Modulus (MPa)	Bulk Modulus (MPa)
1950	2.4	0.22	5500	13,420	7988.1

**Table 2:** Natural frequency of the WTB.

Order	1	2	3	4	5
Frequency (Hz)	7.32	18.89	27.61	58.16	82.73

**Table 3:** The characteristics of the FBGs.

Central Wavelength (nm)	Gating Length (mm)	Bandwidth (mm)	Reflectivity (%)	Side-Mode Suppression Ratio (dB)
1550	2.0	0.45	69.3	10.38
